# CRISPR/Cas9-Based Deletion of SpvB Gene From *Salmonella gallinarum* Leads to Loss of Virulence in Chicken

**DOI:** 10.3389/fbioe.2022.885227

**Published:** 2022-06-13

**Authors:** Abdul Basit, Hamza Tahir, Zulquernain Haider, Hafsa Tariq, Asim Ullah, Shafiq Ur Rehman

**Affiliations:** Institute of Microbiology and Molecular Genetics, University of the Punjab, Lahore, Pakistan

**Keywords:** CRISPR/Cas9, *Salmonella gallinarum*, fowl typhoid, poultry, virulent plasmid, SpvB

## Abstract

*Salmonella* Gallinarum causes fowl typhoid in poultry leading to a huge economic loss to the poultry industry. The large virulence plasmid of *S. gallinarum* has been associated with various systemic infections in poultry. A five-gene spanning region (spv*RABCD*) of 7.8 kb on the large plasmid mainly confers virulence to the bacteria. However, the exact role of these genes in virulence has not been elucidated yet. SpvB exhibits delayed cell death by preventing actin polymerization followed by apoptosis during intracellular infection. The specific role of SpvB in causing the disease is not known yet. In the current study, the SpvB gene was deleted through CRISPR/Cas9 method from a large virulent plasmid of locally isolated *S. gallinarum* strain (SG18). The homology-directed repair method was used for complete deletion of SpvB gene using the modified pCas9 plasmid. The SpvB-deleted *S. gallinarum* strain (ΔSpvB_SG18), when tested for its virulence in broiler chicken showed no diseases signs and mortality. In addition, the avirulent strain does not affect the bird’s weight and was rapidly cleared from the liver after infection. However, it cleared from the intestine only after 4–5 days, which suggests that the ΔSpvB_SG18 strain is unable to invade from the intestine to the liver. This is the first study to report a complete gene deletion from the *S. gallinarum* virulent plasmid and its effect. This method will be useful for the deletion of virulent genes from *S. gallinarum*, to study their role in pathogenesis, and to prepare an effective vaccine strain for controlling fowl typhoid in poultry.

## Introduction

Fowl typhoid is a severe, systemic disease of poultry caused by *Salmonella enterica* subsp. *enterica* serovar gallinarum biovar gallinarum (Shivaprasad et al., 2008). Although, *S. gallinarum* has been eradicated in developed countries, it is still a problem in developing countries (Andino and Hanning, 2015) and leads to a massive loss of poultry flocks (Mamman et al., 2014; Geetha and Palanivel, 2018). Pakistan’s poultry industry contributes 34% to total meat production (Islamic Markets, 2019), with a significant contribution to the national GDP (1.3%) (Hussain et al., 2015). Various virulence factors of *S. gallinarum* are claimed to be responsible for causing fowl typhoid; nevertheless, less is known about the genetics of *S. gallinarum*’s pathogenicity (Li et al., 1993). Virulent factors in enteric bacteria are often encoded by plasmids; however, the exact role of the virulent plasmids in pathogenesis is not clear yet (Singh et al., 2018). Previously, the large virulent plasmid (85 kb) of *S. gallinarum* has shown to be mainly involved in causing systemic infection in poultry; however, the exact function of the virulent genes in their pathogenicity has not been elucidated yet (Barrow et al., 1987). The *spv* locus on the large virulent plasmid harboring five genes (spv*RABCD*)*,* is highly conserved among *Salmonella* serovars and is sufficient to restore the virulence, hence causes mortality in birds. However, the sequencing of spv genes has provided no clues about their function (Rotger and Casadesús, 1999; Jones et al., 2001). Although, the SpvB mutant of *Salmonella dublin* was found avirulent form in mice (Roudier et al., 1992), while another experiment has shown that SpvB together with SpvC is sufficient to confer the virulence to *Salmonella typhimurium* in mice (Käppeli et al., 2011; Kidwai et al., 2013). However, the specific role of the SpvB gene alone in causing the disease in poultry is not determined yet (Cheng and Wiedmann, 2019), as compared to other plasmid-encoded virulent genes, particularly considering the previous studies showing that both SpvB and SpvC are essential for mediating the toxin-associated phenotype *in vivo* (Matsui et al., 2001a; Guiney and Fierer, 2011). On the other hand, the serovars gallinarum does not cause systemic diseases in experimentally infected mice or other laboratory mammals (Barrow et al., 1994), which is an important exception. Therefore, the virulent role of SpvB in *S. gallinarum* in causing poultry infection needs to be determined.

CRISPR/Cas9 is an efficient tool for gene editing of bacterial genomes. However, the success rate of genome editing in bacterial plasmids through CRISPR/Cas9 is very low, as a single unedited plasmid (if remaining) can make their replicas, thus restoring their natural sequences (Tagliaferri et al., 2020). Recently, the CRISPR/Cas9 system has been applied for the deletion of virulent genes from genomic DNA (Jiang et al., 2013) or the curing of virulent plasmids (Wang et al., 2019; Tagliaferri et al., 2020), from clinical pathogens. Here, we describe the modified CRISPR/Cas9 system to delete the plasmid-based SpvB gene from *S. gallinarum*, isolated from local poultry production. The SpvB gene encodes ADP-ribosylating toxins, which destabilize cytoskeletons in host cells, playing an important role in pathogenesis of *Salmonella* spp. (Skyberg et al., 2006). Moreover, 9R strain of *S. gallinarum* (live attenuated) has been used worldwide as a vaccine since 2001; however, due to the persistent evolution of the pathogen, the vaccine strain may not provide sufficient protection against *S. gallinarum* (Lee et al., 2007; Kim et al., 2020). Therefore, the current study aimed to produce an avirulent strain by deleting the SpvB gene from *S. gallinarum* virulent plasmid through the CRISPR/Cas9 system. The deletion mutant was further analyzed for their *in vivo* disease-causing potential in poultry.

## Materials and Methods

### Bacterial Strains, Plasmids, and Chemical Reagents

*S*. *gallinarum* strains were isolated from poultry samples with fowl typhoid symptoms and identified by our laboratory [data not shown]. *E. coli* top 10 cells were used for transformation and propagation of recombinant plasmids. For cloning of the DNA editing template, pETT22b (+) vector was used, while pCasSA (Plasmid #98211) and pCas9 (Plasmid #42876) plasmids purchased from addgene were used for gRNA cloning. Plasmid, genomic DNA, and gel purification kits were purchased from Thermoscientific, United Kingdom. Golden gate (E1601) and HiFi assembly kits (E2621, NEB, New England Biolab) were used for the cloning of gRNA and DNA editing templates, respectively. The chemicals and the media used were purchased from Alpha Biosciences, United States. Sanger sequencing and oligos synthesis were commercially performed from Macrogen, Korea.

### Oligos Designing and PCR Amplification

The gene sequence of SpvB (GenBank ID: D14490.1) was retrieved from NCBI. For targeted deletion of the SpvB gene from a large size plasmid of *S. gallinarum*, gRNA was designed though the CHOP CHOP online tool. The gRNA (OligoI & II) sequence showing high target efficiency was synthesized (Table 1).

**TABLE 1:** Details of the primers used in this study.

Primer	Sequence	Other details
Oligo I	AAA​CGA​TCA​CAG​AGT​CGT​ATA​CCG​G	gRNA (5՛→3՛) targeting SpvB gene, with the BsaI restriction site
Oligo II	CGG​TAT​ACG​ACT​CTG​TGA​TCC​AAA​A	gRNA reverse complement strand (5՛→3՛) targeting the SpvB gene, with the BsaI restriction site
H1F	TTG​AGA​TCT​GTC​CAT​ACC​CAT​GGTCT​AGAGTT​CCG​TTG​CTC​CCC​AAA​CCC​A	Forward primer to amplify homologous arm1 (1 kb upstream region of SpvB gene) having an overlap for the pET22b vector and the XbaI restriction site
H1R	GGC​CAG​TTT​CAG​GAG​ATA​GTG​TAT​ACT​AAG​AAT​CGA​TTC​CAG​AAG​T	Reverse primer to amplify homologous arm1 (1 kb upstream region of the SpvB gene) an having overlap for homologous arm2
H2F	CTT​CTG​GAA​TCG​ATT​CTT​AGT​ATA​CAC​TAT​CTC​CTG​AAA​CTG​GCC	Forward primer to amplify homologous arm2 (1 kb downstream region of the SpvB gene) having an overlap for homologous arm1
H2R	GAT​ACA​GGT​ATA​TTT​TTC​TGA​CTC​GAG​TTC​ACA​GGT​CGT​AAC​CGC​CAT​CC	Reverse primer to amplify homologous arm2 (1 kb downstream region of the SpvB gene) having an overlap for the pET22b vector
C1	ATA​GTG​ACT​GGC​GAT​GCT​GTC	Reverse primer for gRNA confirmation in pCas9. This primer is used in combination with OligoI and amplifies a 180 bp fragment
C2	ATG​GGT​ATG​GAC​AGA​TCT​C	Reverse primer for gRNA confirmation in pCasSA when used in combination with OligoI and amplifies 130 bp fragment
Screen_F	GGA​ATT​CGT​CAG​TAA​GGG​GGG​A	Forward primer binds to 1.1 kb upstream of the SpvB gene
Screen_R	AAC​CGC​GAT​TCC​GCA​CAG​CAG​AA	Reverse primer binds to 1.2 kb downstream of the SpvB gene and amplifies a 4.3 kb fragment from unedited large virulent plasmid of SG18, while 2.2 kb fragment in case of SpvB gene deletion
SpvB_F	CTA​AAT​GGT​TTT​TCA​TCT​GCC​AC	These primers are used for detection of the SpvB gene in *S. gallinarum* strains
SpvB_R	TGT​ACC​TTG​CTG​AGA​TAG​CGC​ATG	

### Preparation of a DNA Editing Template

In order to clone the DNA editing template in the pET22b(+) vector, one kb upstream fragment (homologous arm1) of the SpvB gene was amplified by H1F and H1R as forward and reverse primers, respectively. Similarly, one kb fragment (homologous arm2) downstream of the SpvB gene was amplified by H2F and H2R as forward and reverse primers, respectively. The homologous arms were then assembled through the HiFi kit according to the manufacturer’s protocol and the fused product of homologous arms were PCR-amplified through H1F and H2R as forward and reverse primers, respectively, followed by cloning in the pET22b(+) vector (already restricted with XbaI and XhoI), by using the HiFi assembly kit. The HiFi assembly mixture was electroporated into Top10 electrocompetent cells at 1,700 V for 5.1 ms using Eppendorp eporator (Eppendorp AG, 22,331, Hamburg). Positive transformants were confirmed through colony PCR using H1F and H2R as forward and reverse primers, respectively. The recombinant plasmid containing DNA editing template (pET_HAs) was isolated from the positive transformants and further confirmed through restriction digestion by using XbaI and XhoI and verified by Sanger sequencing.

### Cloning of gRNA in pCas9 and pCasSA Vectors

In order to clone gRNA oligos (OligoI and OligoII) in pCas9 and pCasSA vectors, phosphorylation of the guide RNA oligos were performed, followed by cloning in the corresponding vectors through the golden gate assembly kit (Engler et al., 2009) using *E. coli* Top10 cells. The cloning of gRNA in pCas9 (pCas9-g) and pCasSA plasmid (pCasSA-g) were confirmed through colony PCR using OligoI as forward for both vectors, while C1 and C2 as reverse primers for pCas9-g and pCasSA-g, respectively, and were further confirmed through Sanger sequencing. The sequences of all the primers and oligos are given in Table 1. Details of the recombinant plasmids produced are given in Supplementary Table S2.

### Electroporation of *S. gallinarum* With Recombinant Plasmids

The recombinant plasmids pCas9-g and pET-HAs carrying the gRNA and DNA editing templates, respectively, were co-electroporated into electrocompetent *S. gallinarum* and spread on an agar plate containing chloramphenicol and ampicillin. Similarly, pCasSA-g and pET-HAs electroporated into *S. gallinarum* were spread on kanamycin (60 μg ml^−1^) and ampicillin (100 μg ml^−1^) agar plates, followed by incubation at 37°C overnight. Plasmids without gRNA and DNA editing templates were also electroporated as negative controls.

### Screening of SpvB Gene-Deleted Strains

Randomly, 25 colonies of *S. gallinarum* co-transformed with pCas9-g and pET-HAs plasmids and 20 colonies of *S. gallinarum* transformed with pCasSA-g and pET_HAs plasmids were picked and screened through colony PCR, for the successful deletion of the SpvB gene using Screen_F and Screen_R as forward and reverse primers, respectively (Table 1). These primers were designed to bind 50 bp upstream and downstream regions of the homologous arms flanking the SpvB gene, which allows the specific binding of these primers only with the large virulent plasmid of *S. gallinarum*. The large virulent plasmid of *S. gallinarum* was used as control.

### Animal Trials on Poultry to Evaluate Virulence

*S. gallinarum* wild type and engineered strains (ΔSpvB_SG18) were used to infect chickens. Disease-free male Cobb 500 broiler chickens (*n* = 120) were used in this study. Day old chicks were obtained from a commercial hatchery. The birds were housed in the pre-sterile cages in an environmentally controlled room with a 16-h light period. Birds were vaccinated against ND (new castle disease) and IB (Infectious bronchitis) on the first day and distributed randomly into three groups, SG_WT (*S. gallinarum* positive control), SG_Healthy (not infected with any pathogens), and ΔSpvB_SG18, each with four equal sized replicates containing 10 birds. The birds were screened for *Salmonella* infection on day 7 through cloacal swabs or fresh fecal samples. Water and antibiotic-free feed were provided to the birds throughout the span of the experiment. At 16 days of age, the birds were inoculated orally with 0.5 ml of normal saline containing approximately 1 × 10^8^ colony-forming units (CFU) of each strain. The birds were monitored for any gross signs and symptoms on a daily basis. The chickens were also weighed three times per week during the experiment time to document any changes in weight gain post infection. Clinical signs and postmortem examinations of the dead birds along with severely ill birds were performed for 3 weeks post infection to evaluate gross pathologies. Post mortem examination of dead and severely ill birds were performed from day 7.

## Results and Discussion

### Role of the SpvB Gene in Virulence

**FIGURE 1:**
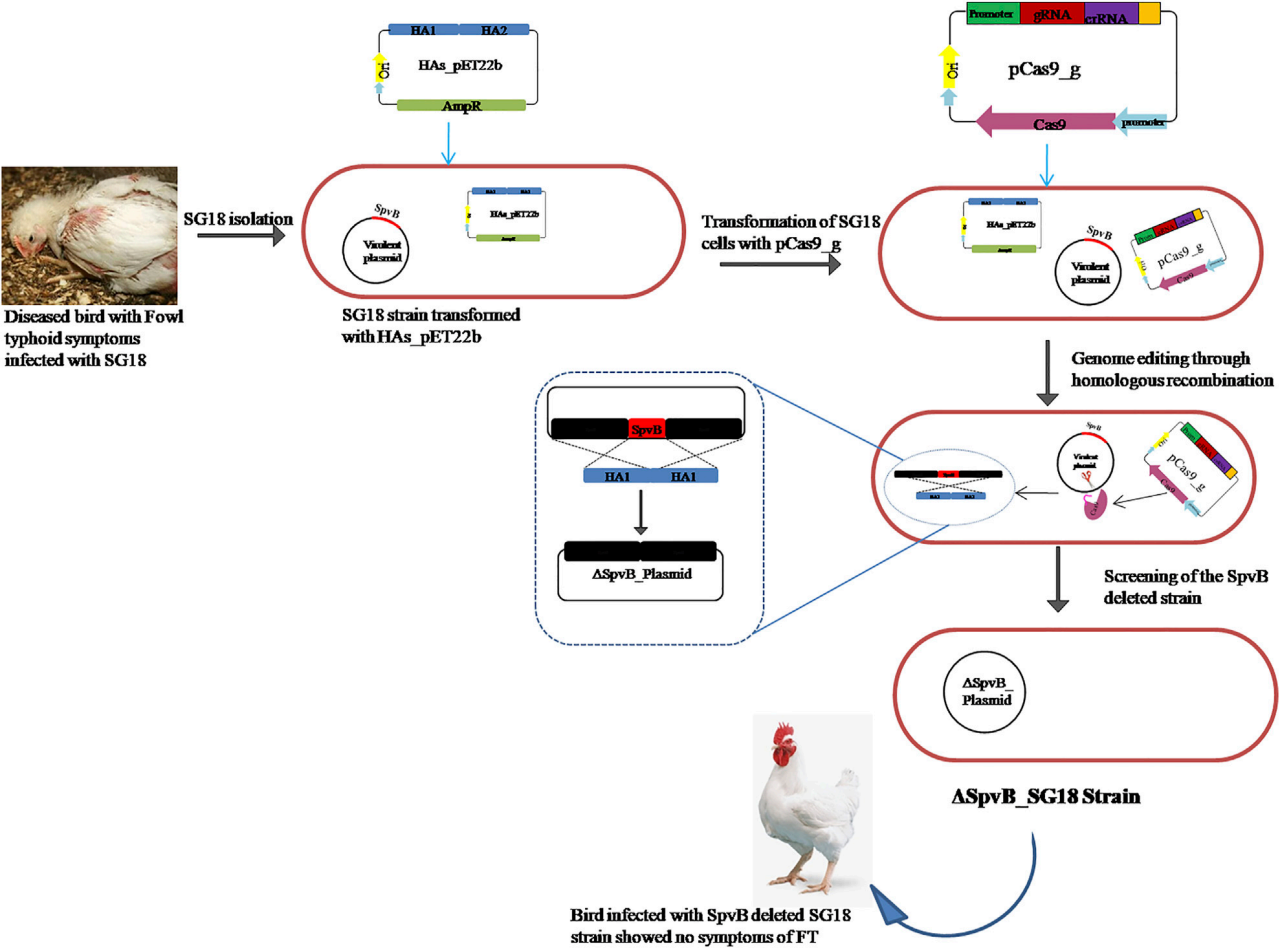
Diagrammatic representation of SpvB gene deletion from *S. gallinarum* large virulent plasmid through engineered CRISPR/Cas9 mediated homologous recombination.

A total of 20 strains of *S. gallinarum* (SG1-20) isolated from poultry samples were screened for possession of the SpvB gene by using SpvB_F and SpvB_R as forward and reverse primers, respectively (Table 1). All the tested strains showed a successful amplification of the SpvB gene (Supplementary Figure S1), indicating the prevalence of the SpvB gene commonly found in *S. gallinarum* isolated from local poultry samples. The majority of *S. gallinarum* virulence genes are distributed in genomic DNA (Asten and Dijk, 2005; Blondel et al., 2010). However, there is no clear evidence available showing a single virulence gene over genome of *S. gallinarum* responsible for mortality in chickens (Shah et al., 2005). Many genes are responsible for the virulence of *S*. *gallinarum* in poultry. A single gene has been shown to reduce virulence significantly in *S*. *gallinarum*. For example, crp, the global transcriptional regulator is associated with virulence and deletion of crp made SG avirulent (Rosu et al., 2007; Mitra et al., 2013). Virulence plasmids are required to trigger systemic disease; however, their involvement in the enteric stage of the infection is unclear. *Salmonella* virulence plasmids are heterogeneous in size (50–90 kb), but they all share a 7.8 kb region, *spv*, required for bacterial multiplication in the reticulo endothelial system. The spv region harbors five genes, *spvR, A, B, C,* and *D*. However, it is still unclear which gene is mainly responsible for virulence, though the SpvB sequence shows a certain degree of similarity to the Ace toxin of *Vibrio cholerae*, which contributes to diarrhea (Trucksis et al., 1993). SpvB and C genes have been reported to cause virulence in *S. typhimurium* in a mouse infection model (Roudier et al., 1992; Matsui et al., 2001b). A recent study has shown that the length of the linker connecting N and C terminal domains of SpvB increases pathogenicity of *S. gallinarum* in chickens (Kim et al., 2020). Nevertheless, there is no evidence available of SpvB’s role in causing FT in poultry. We aimed to explore the role of SpvB gene of *S. gallinarum* in causing fowl typhoid and mortality in chickens, by deleting a complete SpvB gene through the engineered CRISPR/Cas9 system, as shown in the schematic diagram (Figure 1). The SpvB gene sequence retrieved from the large virulent plasmid isolated from *S. gallinarum*, sequenced through NGS, showed >98% homology with the previously reported SpvB gene from SG18 strain of *S. gallinarum and* other *Salmonella* Sp. (Supplementary Figure S2; Supplementary Table S1).

### Genome Editing Plasmid Construction

**FIGURE 2:**
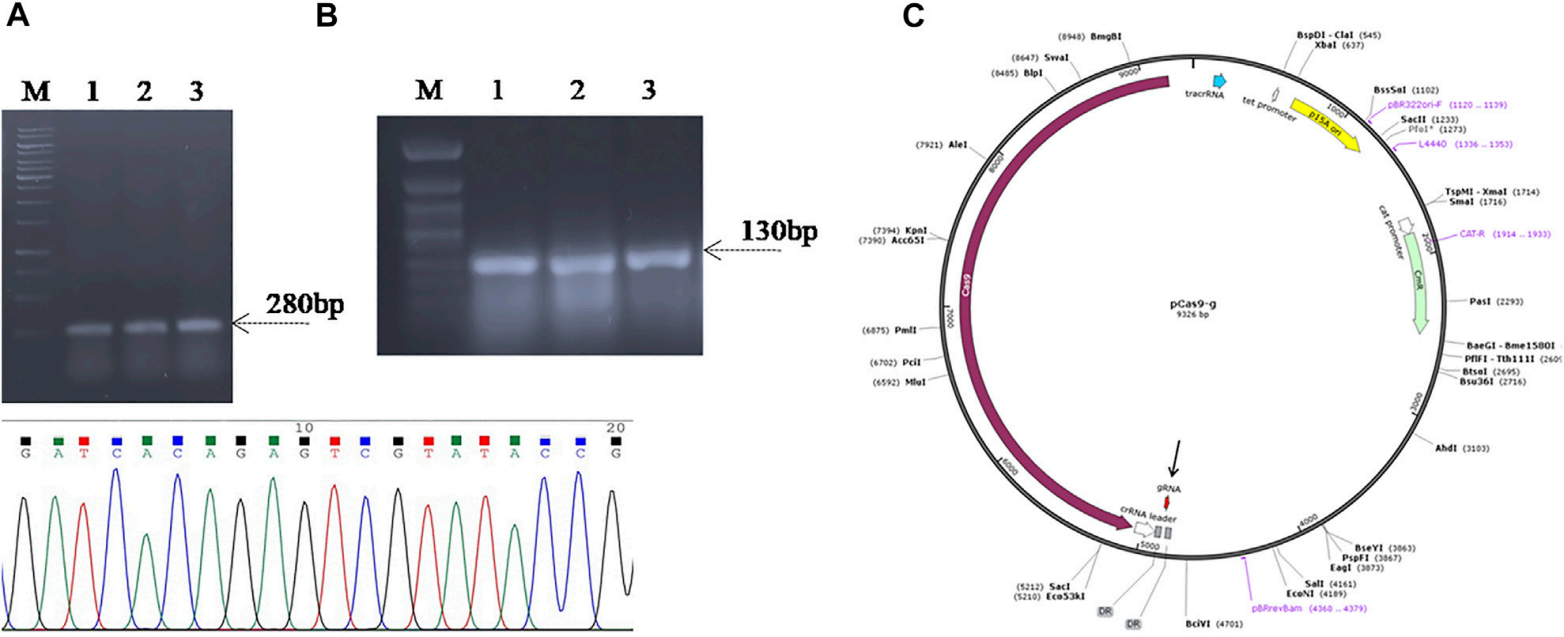
Confirmation of spacer RNA (gRNA) cloning in **(A)** pCas9 **(B)** and pCasSA vectors through PCR and Sanger sequencing. Lanes 1–3, colony PCR of the Top10 cells carrying recombinant pCas9-g showed amplification of a 180 bp fragment suggest the cloning of gRNA in pCas9. Lanes 4–6, colony PCR of the cells carrying the recombinant pCasSA-g plasmid amplified 130 bp fragment suggest the cloning of gRNA in pCasSA, which is further confirmed through Sanger sequencing shown in the chromatogram. **(C)** Plasmid map of recombinant pCas9-g carrying spacer RNA targeting the SpvB gene. The gRNA (colored red) is denoted with an arrow. Lane M, Gene Ruler 1 kb DNA ladder (SM0311), Lane M*, Gene Ruler 50 bp DNA ladder (SM0371) was used as the DNA ladder.

**FIGURE 3:**
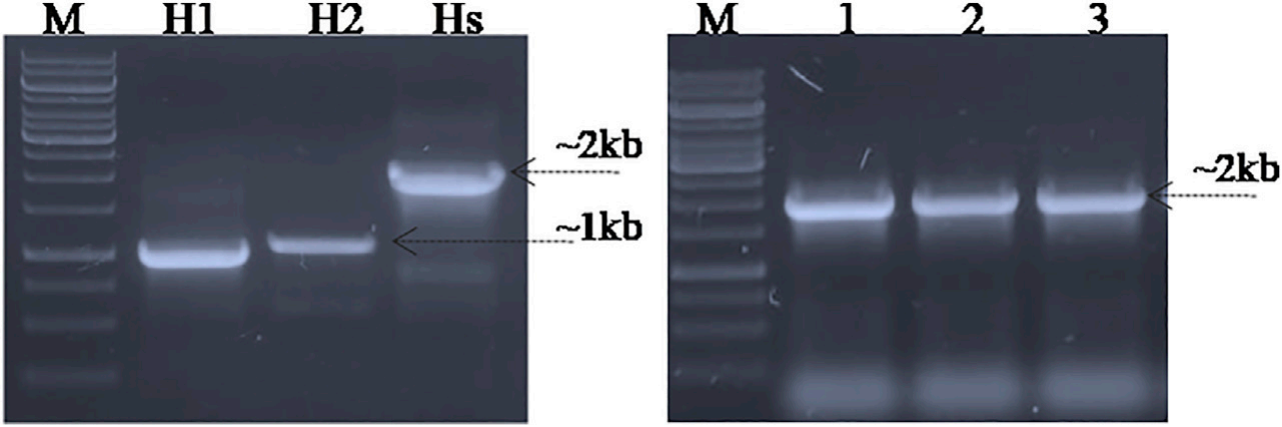
PCR amplification and cloning of homologous arms in the pET22b (+) vector. Lane H1; homologous arm1, Lane H2; homologous arm 2, Lane Hs; fused homologous arms, Lane P; PCR amplified fused homologous arms was used as positive control, Lane 1–3; Colony PCR of Top10 cells carrying fused homologous arms as the DNA editing template cloned in the pET22b (+) vector (pET_HAs).

For targeted deletion of the SpvB gene, a spacer RNA targeting SpvB gene were cloned between two direct repeats in pCas9 plasmid (Jiang et al., 2013) and the plasmid was named as pCas9_g (Figure 2C). Similarly, the gRNA was also cloned in the spacer insertion site of pCasSA plasmid under cap1A promoter (Chen et al., 2017) and named as pCasSA_g (Supplementary Figure S3). The expression of the Cas9 protein in pCasSA plasmids was driven by strong rpsL promoters (Cobb et al., 2015). Cloning of gRNA in the respective plasmids was confirmed through PCR and Sanger sequencing (Figures 2A,B). The DNA-editing template containing homologous arms flanking the SpvB gene were used for homologous recombination mediated repair of the double strand DNA break (Figure 3). The gRNA was cloned through the one-step golden gate assembly reaction (Engler et al., 2009), while homology arms were cloned through the one-step HiFi assembly cloning reaction (Gibson et al., 2009) in their respective plasmids. Since, the pCas9 promoters are derived from Gram-negative bacteria, while pCasSA promoters are derived from Gram-positive bacteria, therefore, pCas9 editing efficiency may be higher in *S. gallinarum* than the pCasSA, while, due to the reduced expression of gRNA and Cas9 protein, the editing efficiency of pCasSA_g may be lower.

### Electroporation of *S. gallinarum* With Recombinant Plasmids

In order to delete the SpvB gene from the large virulent plasmid of *S. gallinarum*, the SG18 strain was co-electroporated with the pCas9_g and pCasSA_g vectors, in combination with the pET_HAs vector. The transformed cells showed growth on an agar plate containing all respective antibiotics. The gRNA directs the Cas9 endonuclease to the SpvB gene locus that is adjacent to a PAM, which produces a double-stranded nick at the gRNA bound locus of the plasmid genome. The cell repair machinery repairs the double-stranded break. However, the presence of DNA editing templates delete the targeted SpvB gene by integrating the homologous arms flanking the SpvB gene through homologous recombination (Chen et al., 2017). Furthermore, to test the toxicity of pCas9, empty pCas9 and pCasSA plasmids were electroporated into SG18 cells, which resulted in more than 300 colonies per plate, suggesting no off-target cleavage of bacterial genomes. More than 100 colonies were obtained after electroporation with the pCas9_g plasmids, while >200 colonies were obtained when the cells were electroporated with the pCasSA_g plasmid, suggesting a lower DNA editing efficiency of the pCasSA-g plasmid in *S. gallinarum*.

### Screening for SpvB Gene Deletion in *S. gallinarum*

**FIGURE 4:**
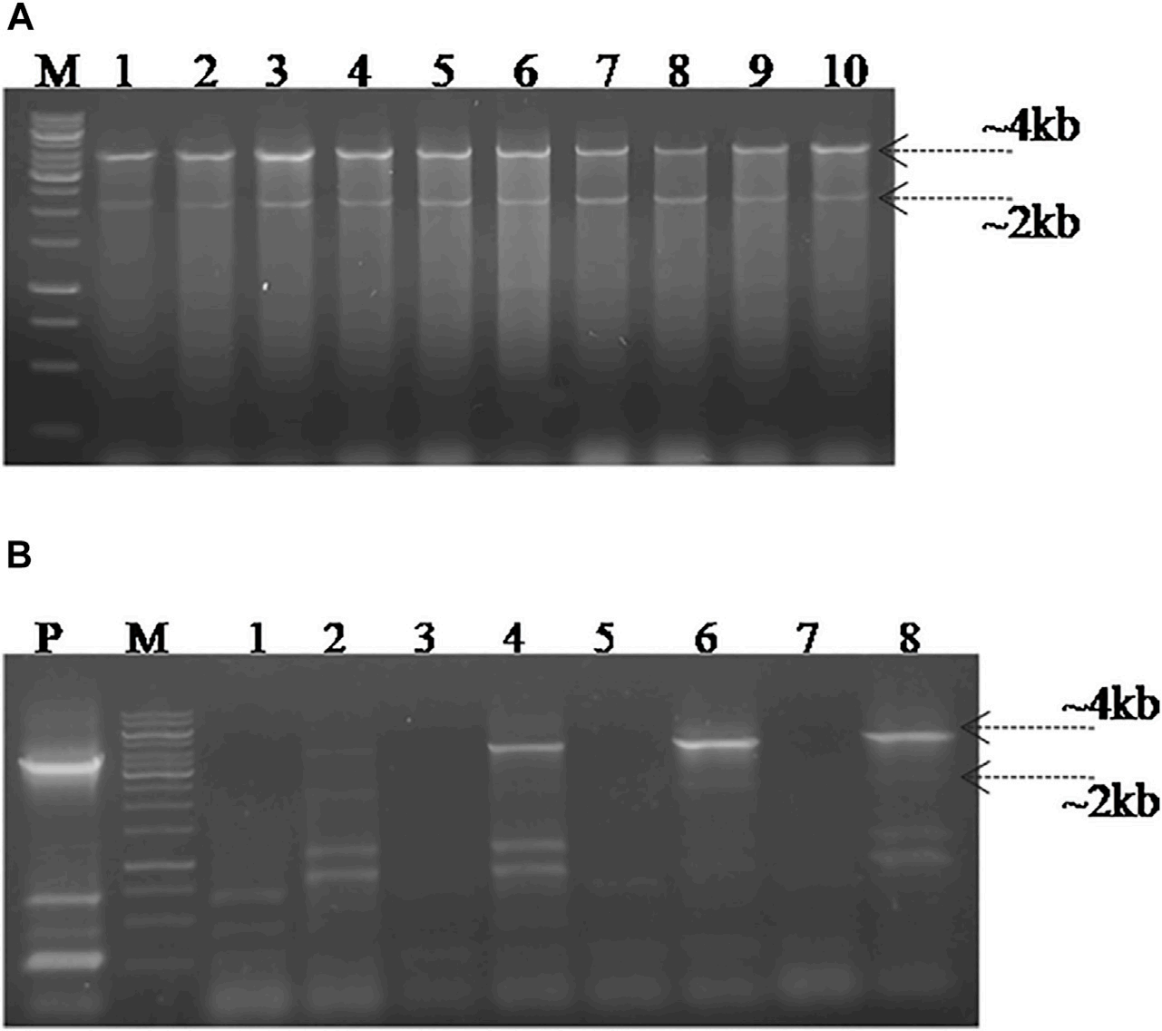
Screening for SpvB deletion in SG18 cells co-transformed with pCas9_g and pCasSA_g plasmids in combination with the DNA editing template carrying vector. **(A)** All the pCas9-g transformed SG18 colonies screened showed a partial deletion of the SpvB gene, while **(B)** pCasSA-g transformed SG18 cells showed a partial deletion of the SpvB gene in 40% of total cells screened. Lanes 1–10 are the colonies electroporated with corresponding vectors, Lane P; large virulent plasmids isolated from SG18 cells were used as positive control, showing amplification of the SpvB gene (4 kb) through screening primers.

**FIGURE 5:**
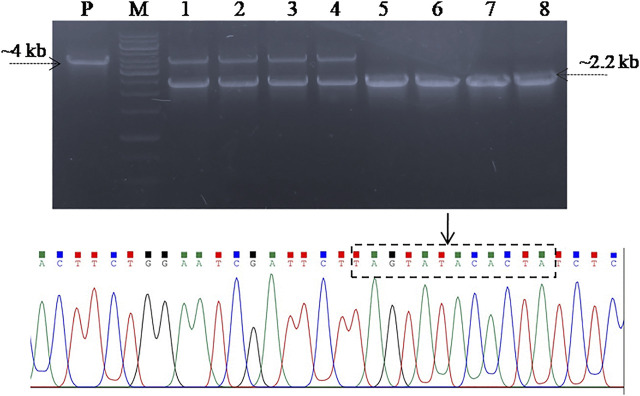
Screening SG18 cells for SpvB gene deletion. The cells were primarily electroporated with the pET_HAs vector and after confirmation, subsequently; the DNA editing template-carrying cells were electroporated with pCas9_g vector. PCR-based screening showed that almost 50% cells screened showed a complete deletion of the SpvB gene, while the rest showed a partial deletion as shown in the aforementioned figure. The deleted mutant further confirmed through Sanger sequencing showed successful deletion of the SpvB gene from the large virulent plasmid of *S. gallinarum*, as shown in the aforementioned chromatogram. The encircled area (denoted by arrow) showing fused homology arms1 and 2 with no SpvB gene.

In order to screen the SpvB gene deletion in pCasSA_g and pCas9_g transformed cells, colony PCR was performed using SpvB screening primers. The cells transformed with pCasSA_g and pET_HAs plasmids showed partial deletion of the SpvB gene in 10/25 colonies. Similarly, cells transformed with pCas9_g and pET_HAs plasmids also showed partial deletion of the SpvB gene in 25/25 colonies (Figure 4). The partial deletion may be due to either a low copy number of the DNA editing template or a rapid replication of uncut plasmids. To increase the copy number of the DNA editing template, we first electroporated the SG18 cells with the DNA editing template and confirmed that the isolate carrying pET_HAs plasmid were subsequently electroporated with pCas9_g*.* The colonies appearing showed 13/25 partial deletion while 12/25 showed a complete deletion of the SpvB gene, confirmed through colony PCR (Figure 5). This 48% complete deletion efficiency was obtained due to subsequent electroporation of DNA-editing plasmids followed by gRNA plasmids. The Sanger sequencing further confirmed that the SpvB gene was completely deleted in the ΔSpvB_SG18 strain, with no mutation in the up and downstream regions of the deleted gene, suggesting that SpvC and A were intact and should be functional in the ΔSpvB_SG18 strain (Supplementary Figures S6, S7). Interestingly, the SG18 cells co-electroporated with two spacer RNAs (pCas9_g and pCas9_g*) targeting distinct regions in the SpvB gene did not show any amplification through the colony PCR (Supplementary Figure S4), which suggests that the double-stranded break produced at two distinct positions in the SpvB gene may not induce effective recombination to achieve the double-stranded break repair, hence it degraded the plasmid (Wang et al., 2016). However, the plasmid isolated from the same colonies showed SpvB amplification, which suggests that, the uncut plasmid replicated to restore their copies, which is contrary to a previous study that claimed gene deletion from plasmid was confirmed through negative colony PCR (Liu et al., 2020). Our results suggest that screening the bacterial colonies for plasmid editing solely through colony PCR may give false-positive results. We further cured the virulent plasmid from SG18 cells, after subsequent rounds of overnight incubation at 42, 43, 44, and 45°C. The plasmid-cured strain was also tested for its virulence in chickens.

### Effect of SpvB Deletion on *S. gallinarum* in Mediating Virulence in Chickens

**FIGURE 6:**
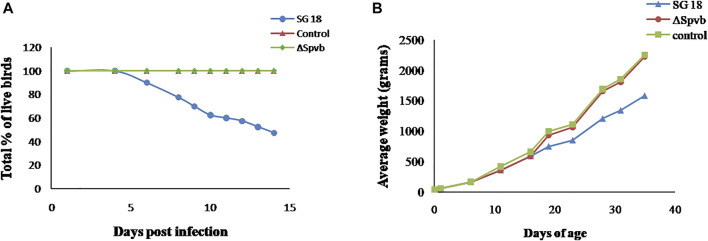
**(A)** Rate of mortality in chickens observed during 15 days after infection with SG18 and ΔSpvB_SG18 strains. **(B)** Effect of SpvB deletion from *S. gallinarum* on poultry weight during the 36 days of experiment.

**FIGURE 7:**
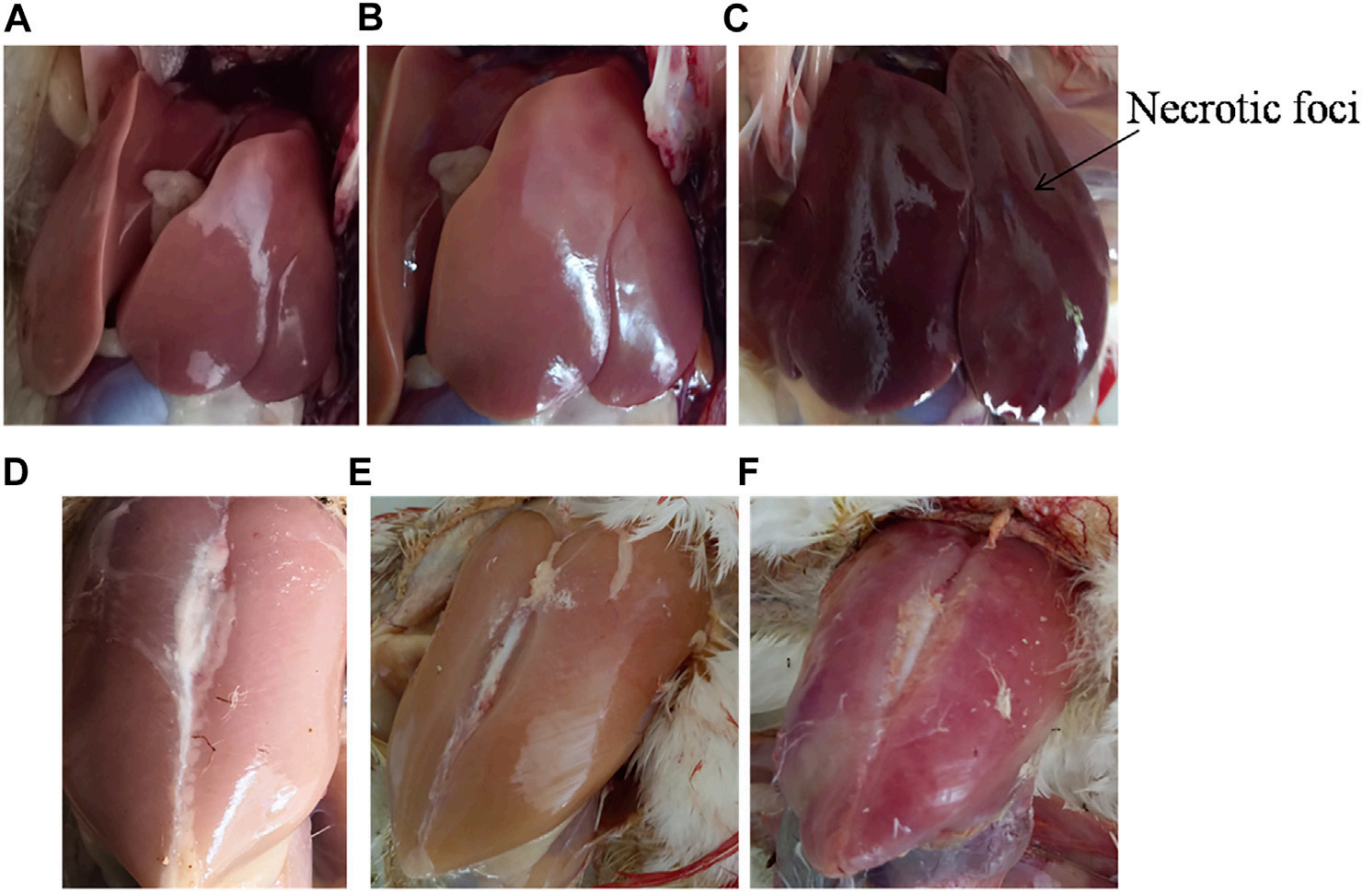
Gross pathology of dead poultry birds exhibiting typical lesions of fowl typhoid. **(A)** Liver of control and **(B)** ΔSpvB_SG18 infected birds showing no signs of fowl typhoid. In contrary, **(C)** the wild type strain (SG18) infected birds showing enlarged livers with necrotic foci, which are typical features of fowl typhoid. Similarly, the gross pathology of **(D)** the control and **(E)** ΔSpvB_SG18-infected birds showed no apparent lesion of disease, suggesting that these chickens were clinically healthy, while **(F)** the SG18-infected birds showed signs of fever and inflammation caused by *S. gallinarum*.

We further determined the effect of SpvB gene deletion from SG18 cells on chicken mortality upon infection. No symptoms of the fowl typhoid disease and mortality were observed in the chickens infected with the ΔSpvB_SG18 strain throughout the 36 days of experimentation, which are in agreement with a previously reported study showing that mutations in SP-2 of *S. gallinarum* resulted in a loss of virulence in chickens (Jones et al., 2001). However, a total of 52% mortality rate was observed in the group infected with the wild-type SG18 strain after 36 days of post infection (Figure 6), which agreed with previous reports showing 50% mortality caused by *S. gallinarum* in domestic fowls (Shivaprasad, 2000). Necropsy of dead birds along with severely ill birds infected with SG18 showed classical signs of fowl typhoid (Figure 7) such as anorexia, lethargy, yellowish diarrhea with pasting, liver enlargement (Saha et al., 2012), catarrhal enteritis with splenomegaly (Shivaprasad, 2000), and pericarditis characterized by thickening of pericardium due to fibrinous exudations along with an enlarged spleen were evident (Supplementary Figure S5). Hemorrhagic edematous lungs with a peculiar yellow color were also a common finding in the birds infected with SG18 (Kumari et al., 2013). In contrary, ΔSpvB_SG18 and virulent plasmid-cured SG18-infected chickens showed no mortality or morbidity with no gross pathological lesions and signs of fowl typhoid throughout the experiment. Interestingly, the weight gain of the birds that survived after the challenge with ΔSpvB_SG18 cells was almost similar to the negative control group pointing out the non-pathogenic behavior of the ΔSpvB_SG18 strain*,* while birds challenged with wild-type SG18 cells showed a marked decrease in weight gain (Figures 6A,B).

### SpvB-Deleted Strains Are Rapidly Cleared After Infection

In order to determine the systemic infection, the infected strains were isolated from visceral organs of the birds, primarily liver and ileum, after 7 and 14 days post infection. The SG18 strain recovered from the liver and intestine of infected birds (positive control group) showed 3.0 × 10^5^ (log10 = 5.4) and 1.6 × 10^8^ (log 10 = 8.2) CFU/g after 7 and 14 days, respectively, post infection. Our results are in agreement with the previously reported study showing the highest number of viable counts that were isolated from the liver after 14 days post infection with *S. gallinarum* (Alves Batista et al., 2018)*.* In contrary, no *Salmonella* were recovered from ΔSpvB_SG18 infected liver and ileum samples, after 4, 7, and 14 days post infection. However, very low counts were obtained from the intestine after 4 days of post infection, suggesting their rapid clearance after infection. Our results suggest that the non-virulent strains (ΔSpvB_SG18) were unable to invade from the intestine to the liver, which is contrary to previously reported avirulent *SsaU* mutant of *S. gallinarum*, found slightly invasive from the intestine to the liver (Jones et al., 2001). A complete deletion of the SpvB gene from *S. gallinarum* resulted in attenuation *in vivo*; however, further deletion of other virulent genes from the ΔSpvB_SG18 strain will make this strain a suitable candidate for vaccine design.

## Conclusion

These results established an appropriate method for the complete deletion of virulent genes from plasmid DNA through CRISPR/Cas9, to produce an attenuated strain of *S. gallinarum*. The SpvB deleted strain of *S. gallinarum* was found completely avirulent in chickens, with no effect on chicken growth rate and weight. This is the first report showing gene editing in *Salmonella* Sp. through CRISPR/Cas9. The procedure opens a path for deletion of other virulent genes from the *S. gallinarum* genome to make the strain completely avirulent. The attenuated strain can then be used as a vaccine for controlling fowl typhoid disease in poultry.

## Data Availability

The original contributions presented in the study are included in the article/Supplementary Material; further inquiries can be directed to the corresponding authors.
