# Supplementary material for: CRISPR/Cas9-Based Deletion of SpvB Gene From Salmonella gallinarum Leads to Loss of Virulence in Chicken
Source: Front Bioeng Biotechnol. 2022 Jun 13;10:885227. doi: 10.3389/fbioe.2022.885227 (PMC9234527; doi:10.3389/fbioe.2022.885227)
Supplement: Supplementary file 1 [file DataSheet1.docx]

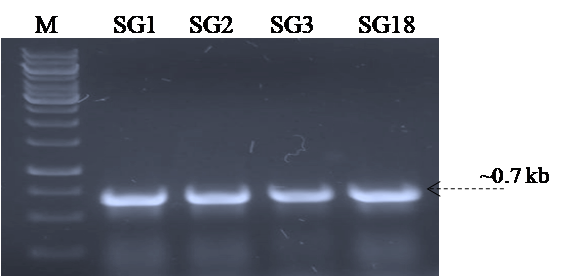


**Figure S1:** Amplification of SpvB gene fragment of ~0.7 kb from *S. Gallinarum* large virulent plasmid in all the tested strains including SG18. (Only SG1, 2, 3 and SG18 strain are shown here, the rest have given same results). Gene Ruler 1 kb DNA ladder (Thermoscientifice, SM0311) was used as DNA ladder.


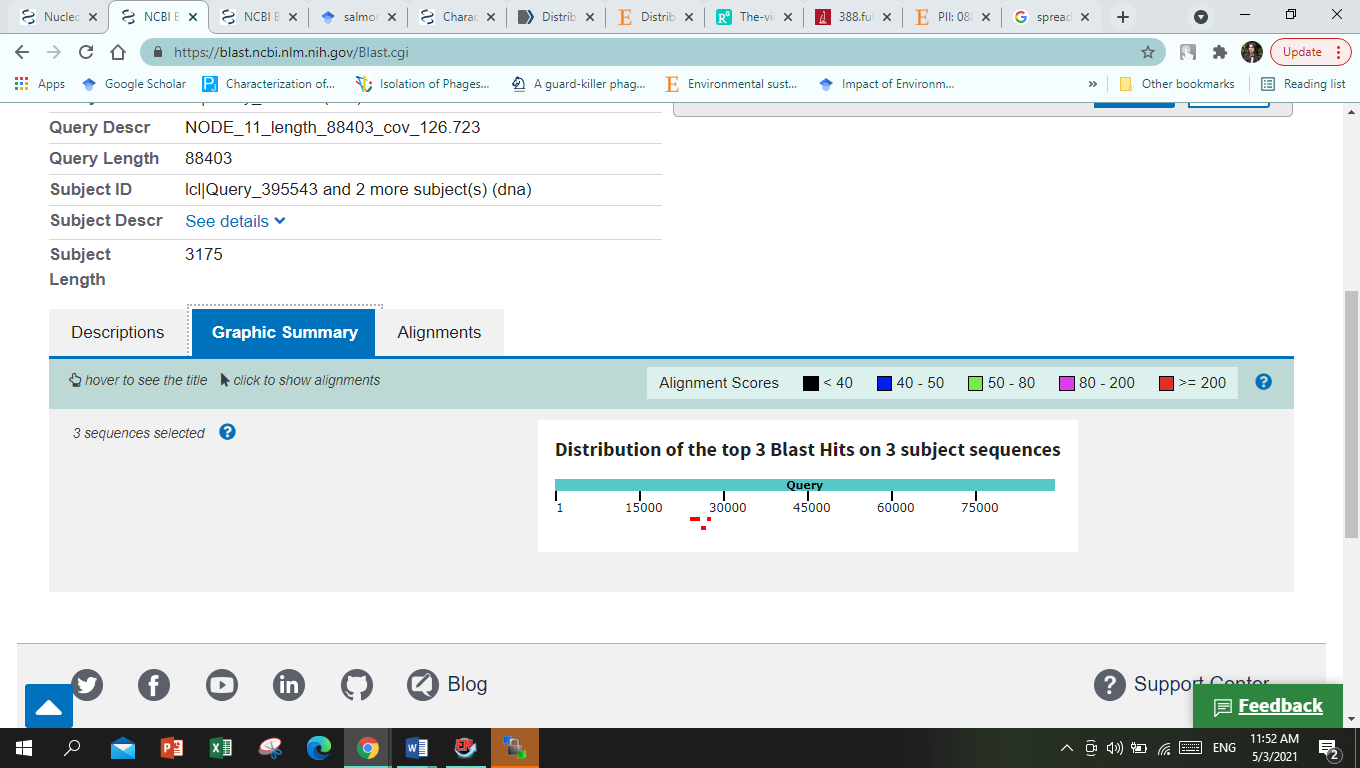


**Figure S2:** Graphical summary of node 11 alignment to spv B, C and D Where red lines indicates for the presence and placement of spv B, C and D genes loci along the length of node 11 shown in blue color.

**
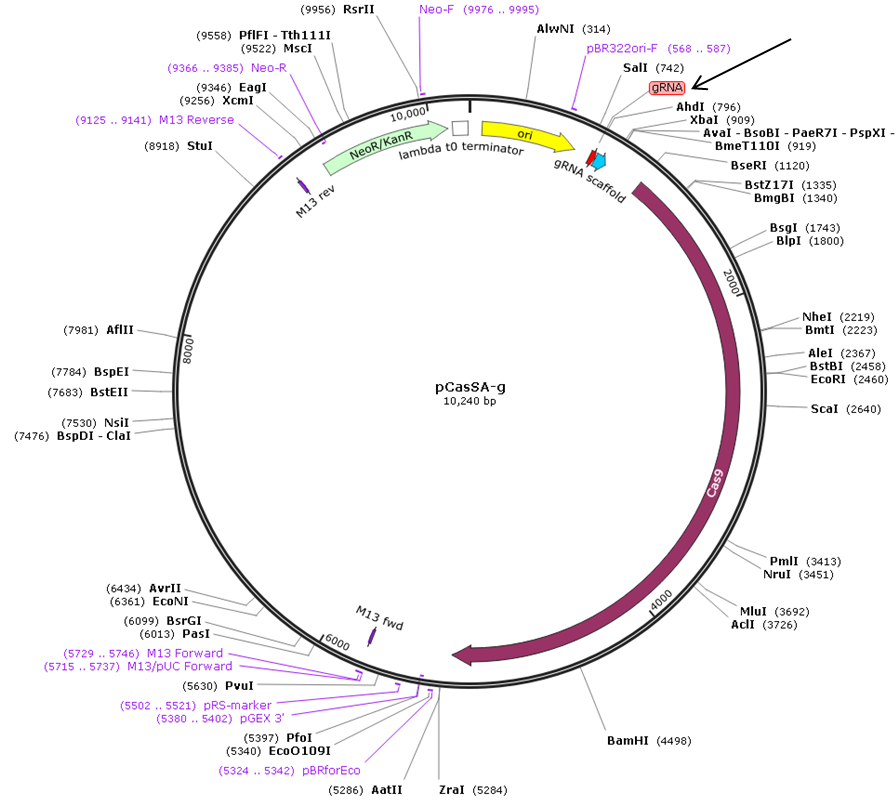
**

**Figure S3:** Plasmid map of the pCasSA-g carrying spacer RNA targeting SpvB gene. The Spacer RNA (gRNA) targeting SpvB gene (colored red) is denoted with an arrow.

**
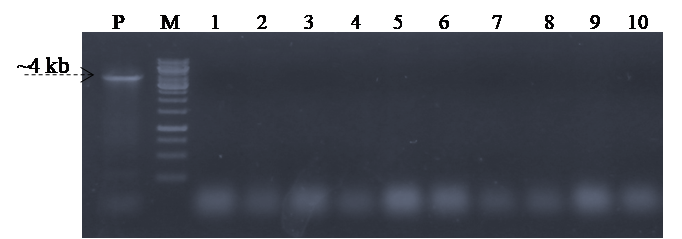
**

**Figure S4**: Screening of SG18 cell for SpvB deletion. The cells were co transformed with two spacer RNAs (pCas9_g and pCas9_g*) cloned in pCas9 plasmids. These two spacer RNAs targeting two different regions of SpvB gene. There is no amplification of either intact or deleted SpvB gene, suggesting degradation of the virulent plasmid after cutting with two different gRNA at two distinct positions.

**
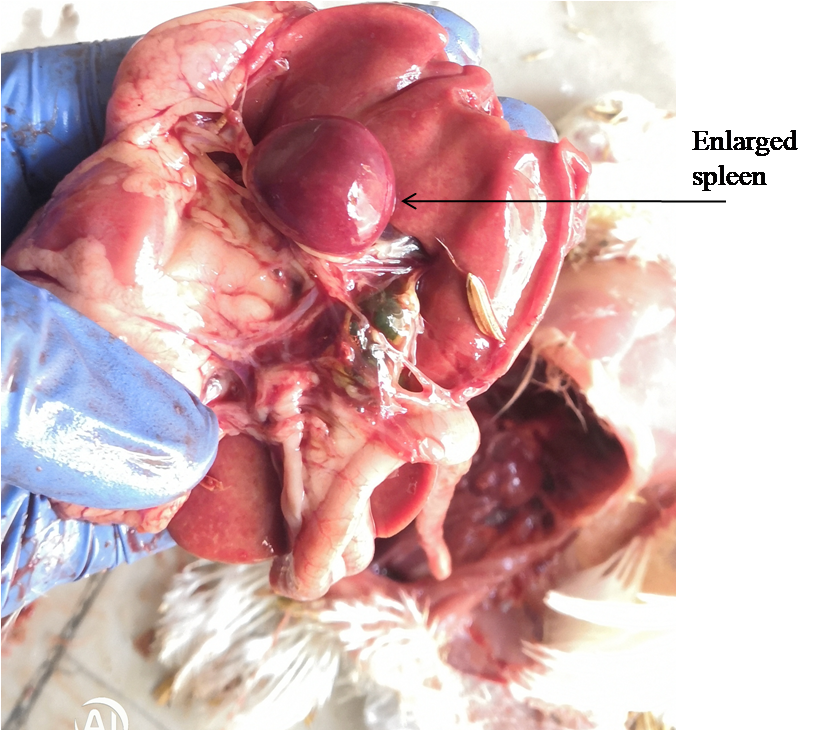
**

**Fig S5**: Post mortem examination of dead birds (infected with SG18) indicating splenomegaly. Enlargement of spleen is indicated with arrow.

**Table S1:** Whole genome assembly and screening of large virulence plasmid after identification of spv genes

| Bacterial strains | Total nodes sequenced | Node#aligned against spv genes | Node length (bp) | % identity to spvB | % identity to spvC | % identity to spvD |
| --- | --- | --- | --- | --- | --- | --- |
| SG-F | 26 | 11 | 88403 | 98.67% | 100.0% | 98.77% |
| SG-4 |  | 11 | 88403 | 98.67% | 100.0% | 98.77% |
| SG-18 | 30 | 11 | 88403 | 98.67% | 100.0% | 98.77% |
| SG-67 | 24 | 11 | 88403 | 98.67% | 100.0% | 98.77% |

**Table S2:**

| **Name of the Plasmid produced** | **Origin of the plasmid** | **Size** | **Resistant marker** | **Features** |
| --- | --- | --- | --- | --- |
| pCas9-g | pCas9 | 9 kb | Chloramphenicol | *S.* Gallinarum genome editing vector used previously for *E. coli*, carrying Cas9 encoding gene, tracrRNA and direct repeats for crRNA cloning. This vector carry gRNA1 targeting SpvB gene |
| pCasSA-g | pCasSA | 10 kb | Kanamycin and Chloramphenicol | Genome editing vector used previously for S. aureus genome editing. This plasmid carrying Cas9 encoding gene, gRNA scaffold with BsaI restriction site for cloning of crRNA. |
| pCas9-g* | pCas9 | 9 kb | Chloramphenicol | pCas9 derivative for SpvB deletion  carrying gRNA2 targeting SpvB gene |
| pET-HAs | pET22b (+) | 6.5 | Ampicillin | Cloning vector carrying DNA editing template of 1 kb up- and downstream of SpvB gene. |

**Figure S6:** One kb upstream region of SpvB gene sequenced from SpvB deleted large virulent plasmid isolated from ΔSpvB_SG18 strain. The sequenced region includes SpvC gene showing no amino acid mutation.

**Figure S7:** One kb downstream region of SpvB gene sequenced from SpvB deleted large virulent plasmid isolated from ΔSpvB_SG18 strain. The sequenced region includes complete cdc of SpvA gene showing no amino acid mutation.
